# First detection of PCV4 in swine in the United States: codetection with PCV2 and PCV3 and direct detection within tissues

**DOI:** 10.1038/s41598-024-66328-y

**Published:** 2024-07-05

**Authors:** Molly Kroeger, Diana S. Vargas-Bermudez, Jairo Jaime, Julian Parada, Jennifer Groeltz, Philip Gauger, Pablo Piñeyro

**Affiliations:** 1 Veterinary Diagnostic Laboratory, Department of Veterinary Diagnostic and Production Animal Medicine, Iowa State University, 1655 Veterinary Medicine, Ames, IA 50011 USA; 2 Animal Health Department. Center of Infectious Diseases and Veterinary Immunology, College of Veterinary Medicine and Production Animal, Colombia National University, Bogotá, Colombia; 3 CONICET- Animal Pathology Department. Agronomy and Veterinary College, Río Cuarto National University, Córdoba, Argentina

**Keywords:** PCV4, In situ hybridization, Lymphoid tissue, Coinfection, PCV2, PCV3, Infectious-disease diagnostics, Infection

## Abstract

Since PCV4 was first described in 2019, the virus has been identified in several countries in Southeast Asia and Europe. Most studies have been limited to detecting PCV4 by PCR. Thus, PCV4 has an unclear association with clinical disease. This study utilized 512 porcine clinical lung, feces, spleen, serum, lymphoid tissue, and fetus samples submitted to the ISU-VDL from June–September 2023. PCV4 was detected in 8.6% of samples with an average Ct value of 33. While detection rates among sample types were variable, lymphoid tissue had the highest detection rate (18.7%). Two ORF2 sequences were obtained from lymphoid tissue samples and had 96.36–98.98% nucleotide identity with reference sequences. Direct detection of PCV4 by RNAscope revealed viral replication in B lymphocytes and macrophages in lymph node germinal centers and histiocytic and T lymphocyte infiltration in the lamina propria of the small intestine. PCV4 detection was most commonly observed in nursery to finishing aged pigs displaying respiratory and enteric disease. Coinfection with PCV2, PCV3, and other endemic pathogens was frequently observed, highlighting the complex interplay between different PCVs and their potential roles in disease pathogenesis. This study provides insights into the frequency of detection, tissue distribution, and genetic characteristics of PCV4 in the US.

## Introduction

Porcine circoviruses (PCVs) belong to the *Circovirus* genus within the *Circoviridae* family, characterized by non-enveloped virions containing single-stranded circular DNA genomes of 1759–2000 nucleotides (nt)^1,2^. Among the four genotypes of PCVs, PCV2 stands out as the predominant pathogen associated with porcine circovirus-associated diseases (PCVAD)^3^, contrasting with the non-pathogenic nature of PCV1^4^. PCV3, discovered in the United States (US) in 2015 through metagenomic sequencing, has been associated with diverse clinical manifestations, including porcine dermatitis and nephropathy syndrome (PDNS), multysitemic inflammatory syndrome, reproductive failure, and subclinical infection^5^. Meanwhile, the emergence of Porcine circovirus 4 (PCV4), initially identified in China in 2019^6^ and subsequently detected in Mongolia^7^, South Korea^8^, Thailand^9^, and Malaysia^10^, has raised interest due to its association with severe respiratory distress and PDNS in pigs. However, the clinical role of PCV4 remains debated^11^, particularly given its concurrent detection with other pathogens^6,12,13^ like PCV2 or PCV3, implying potential coinfection implications.

Until recently, Asia was the only continent where this novel virus had been reported; studies carried out in South America^14^ and Europe^15^ failed to detect PCV4. However, the first detection of PCV4 in Europe was recently reported in wild and domestic pigs from Mid-South-Western Spain^11^. Retrospective studies conducted in China reported the earliest detection of PCV4 DNA in tissue from 2012^12^ and serum from 2008 was positive for PCV4 antibodies^16^.

The 1,770 nt genome of PCV4 has low nucleotide identities to other PCVs at the whole genome level (43.2–51.5%), but high nucleotide identity to mink circovirus^17,18^. Similar to other circoviruses, the ambisense PCV4 genome contains two major open reading frames (ORF) where ORF1 encodes for the replicase protein (Rep, 891 nt) and ORF2 encodes for the capsid protein (Cap, 687 nt)^18^. The PCV4 ORF2 has low identities compared to PCV1, PCV2, and PCV3 ORF2 sequences (approximately 43.1%, 45%, and 24.5%, respectively)^6^. Several studies have proposed preliminary subtype classification primarily based on amino acid patterns of the Cap protein at residues 27, 28, 212 with the genetic distance between subtypes as 0.05. Subtypes identified include PCV4a-1 (27S, 28R, 212L), PCV4a-2 (27S, 28G, 212L), and PCV4b (27N, 28R, 212 M)^18–20^.

Most studies on PCV4 have focused on the detection of viral DNA using conventional or real time quantitative PCR (qPCR) techniques, yielding a variable positivity rate ranging from 1.6 to 45.39%^7,12^. PCV4 has been detected in various pig tissues including heart, liver, spleen, lung, kidney, lymph node, intestine, and brain^12,21^. Additionally, detection of the virus has occurred in a wide range of ages including suckling, nursery, and finishing pigs in addition to its detection in sows and fetuses^7,8,12,13,18^. However, the pathogenesis of PCV4 is not yet well established. Reported clinical manifestations associated with the detection of PCV4 include PDNS^6,12,22^, postweaning multisystemic wasting syndrome (PMWS)^10,13^, neurological signs^12,13^, diarrhea^6,13^, enteritis^12^, encephalitis^10^, respiratory disease^6,9,13^, and reproductive disorders^8^ as well as subclinical infection^8^. Notably, PCV4 often coexists with other PCVs like PCV2 and PCV3^7,9,12,13,16,19,22–24^, with potential implications for disease severity and pathogenesis. Most recently, in situ hybridization studies revealed the presence of PCV4 nucleic acid in bronchiolar epithelium, lymphocytes and histiocyte-like cells in lymph nodes of grower-finisher pigs reported with respiratory signs and sudden death^9^. Furthermore, PCV4 has been rescued from an infectious clone, however, inoculation in piglets did not result in the development of apparent clinical disease and lesions associated with infection^25^. Therefore, further research is needed to conclusively identify lesions associated with PCV4 infection in the field.

Currently the presence of PCV4 in the US swine population is unknown. Previous studies in southeast Asia have codetected PCV4 with PCV2 and PCV3 within a single sample^7,9,12,13,16,19,22–24^. Furthermore, information on the direct detection of PCV4 within tissues and its association with specific lesions is scarce. Therefore, the objectives of this study were to (1) characterize the detection rate of PCV4 in different sample types from clinical submissions, (2) compare molecular features of US PCV4 ORF2 sequences to reference strains, (3) characterize the codetection rate by direct and indirect methods for PCV2, PCV3, and other endemic viral and bacterial pathogens in PCV4 positive samples, and (4) identify the tissue distribution and immune cell types which facilitate PCV4 replication by direct detection methods. Understanding the prevalence and pathological and clinical changes associated with PCV4 infection, in addition to its association with coinfections is paramount for assessing the impact of this novel virus on the global swine industry.

## Materials and methods

### Selection of sample matrixes and cases

All cases were submitted to the Iowa State University Veterinary Diagnostic Laboratory (ISU-VDL) for routine diagnostic investigation. The ISU-VDL is a National Animal Health Laboratory Network accredited laboratory that receives more than 120,000 cases per year, 75% of which are of porcine origin. Approximately 10,000 porcine cases are submitted for gross and histologic evaluation of tissues. For this study, 512 DNA extracts were obtained from clinical cases submitted to the ISU-VDL during June–September 2023 representing six sample matrixes including lung (*n* = 100), feces (*n* = 100), spleen (*n* = 100), serum (*n* = 100), lymphoid tissue (LT, lymph node or tonsil, *n* = 64), and fetus (*n* = 48). Samples were not selected based on the presentation of a specific clinical sign or excluded from the study based on the identification or absence of the etiological agent ruled as the primary case diagnosis. One sample per case was randomly chosen for the study for each sample matrix. No animal samples specifically for this project were collected; therefore, no ethical approval was requested from the ISU institutional animal care and use committee. All experiments were performed in accordance with relevant guidelines and regulations.

DNA extraction from all sample types was carried out at the ISU-VDL according to standard operating procedure. Tissues were minced using sterile scissors and forceps, transferred to a 50 mL conical tube with Earle’s Balanced Salt Solution (Sigma-Aldrich, St. Louis, MO, USA) to make a 10% weight/volume homogenate, the conical tube was then placed in a Geno/Grinder® (SPEX Sample Prep, Metuchen, NJ, USA) at 1000 rpm for 2 min followed by centrifugation at 4200 × g for 10 min at 4 °C and the resulting supernatant was transferred into a 5 mL snap-cap tube. Fecal swabs were collected from colon and placed immediately in 1 mL phosphate buffered saline (PBS). All swabs were vortexed for 15 s at 4200 × g for 10 min at 4 °C and supernatant was transferred into a 5 mL snap-cap tube. DNA extraction from the tissue homogenate, serum, and fecal samples was performed using MagMAX-96Pathogen RNA/DNA kit (Applied Biosystem, MA, USA) and KingFisher Flex 96 Deep-Well Magnetic Particle Processor (Thermo Fisher Scientific, Waltham, MA) following the manufacturer’s instructions.

### PCV4 qPCR

All samples were tested for the presence of PCV4 as previously described^24^. DNA extracts were used to detect a region of the ORF1-ORF2 of PCV4 using Premix Ex Taq (TaKaRa, Dalian, China) with the forward primer 5′-ATTATTAAACAGACTTTATTTGTGTCATCACTT-3′, reverse primer 5′-ACAGGGATAATGCGTAGTGATCACT-3′, and probe 5′-/56-FAM/-ATACTACAC/ZEN/TTGATCTTAGCCAAAAGGCTCGTTGA/3IABkFQ/-3′. The qPCR was performed (7500 Fast Real-Time PCR System, Applied Biosystems, Foster City, CA) with the following cycling conditions: 95 °C for 30 s, followed by 95 °C for 5 s and 60 °C for 1 min for 40 cycles. One positive (plasmid DNA containing the PCV4 genome) and negative control (nuclease free water) was included for each 96-well plate of samples tested. Samples with cycle threshold (Ct) value of < 37 were considered positive. Cross-reactivity of the PCV4 qPCR to PCV2 and PCV3 was tested by the addition of PCV2 virally extracted DNA and plasmid DNA containing the PCV3 genome at 250 ng per reaction under the specified cycling conditions. No amplification of the PCV2 and PCV3 DNA was observed.

### PCV2 and PCV3 Multiplex qPCR

All samples were tested for the presence of PCV2 and PCV3 according to standard operating procedure for a PCV2 and PCV3 multiplex qPCR at the ISU-VDL. DNA extracts were used to detect the conserved region of the ORF1 of PCV2 and PCV3 using TaqMan Fast Virus 1-step Master Mix (Life Technologies, MA, USA) with specific primers previously described^21^. The ORF1 of PCV2 was detected using forward primer 5′-GACTGTWGAGACTAAAGGTGGAACTGTA-3′ and reverse primer 5′-GCTTCTACACCTGGGACAGCA-3′ with the probe 5′-/56-FAM/-CCCGTTGGAATGGT/3MGBEc/-3′^26^. The ORF1 of PCV3 was detected using forward primer 5’-TGTWCGGGCACACAGCCATA-3’ and reverse primer 5’-TTTCCGCATAAGGGTCGTCTT-3’ with the probe 5’-/5SUN/ACCACAAAC/ZEN/ACTTGGCTC/31ABkFQ/-3’^21^. The qPCR was performed (7500 Fast Real-Time PCR System, Applied Biosystems, Foster City, CA) with the following cycling conditions: one cycle at 50 °C for 5 min, one cycle at 95 °C for 20 s, 40 cycles at 95 °C for 3 s and 60 °C for 30 s. Each 96-well sample plate contained three controls: a PCV2 control consisting of 250 ng PCV2 virally extracted DNA, a PCV3 positive control consisting of 250 ng of the PCV3 whole genome cloned into a plasmid, and negative control consisting of nuclease free water. Samples with Ct values of < 37 for either species were considered positive.

### PCV4 open reading frame 2 sequencing and phylogenetic analysis

The DNA extracts from samples with a PCV4 qPCR Ct value of < 34 were selected for ORF2 sequencing. Q5 high-Fidelity 2X Master Mix (New England Biolabs, Ipswich, MA) was used with Forward Primer 5′-TGAGGGAGGATGGGCAGTTGTATG-3′ and Reverse Primer 5′-CACCACCCACAGATGCCAATCA-3′ to amplify the PCV4 ORF2. The PCR was performed (T100 Thermal Cycler, BioRad, Hercules, CA) with the following cycling conditions: one cycle at 98 °C for 30 s; 35 cycles at 98 °C for 10 s, 64 °C for 30 s, and 72 °C for 30 s; and one cycle at 72 °C for 2 min. Successful amplification of the ORF2 was confirmed by the presence of an ~ 800 bp band on a 1% agarose gel. Bidirectional Sanger sequencing of PCR products was carried out by the Iowa State University DNA Facility utilizing the previously specified primer set used for PCR amplification.

PCV4 ORF2 sequences were obtained from GenBank which includes 73 full-length ORF2 sequences as previously described^10,11^ (Additional data table). Sequences were reverse complemented if applicable. Phylogenetic analyses were conducted using Geneious Prime Version 2023.0.1 (http://www.geneious.com). ORF2 nucleotide sequences were aligned by Geneious Prime alignment (global alignment with free end gaps) with 65% similarity (5.0/-4.0). Phylogeny reconstruction and determination of identity percentages utilized the statistical method of Maximum Likelihood (ML) by PhyML^17^ for the Geneious Prime software with the bootstrap method with 1,000 replications. ORF2 nucleotide sequences were translated using standard genetic code and were aligned using Clustal Omega in Geneious Prime. ORF2 sequences obtained in the present study are available on GenBank with accession numbers PP457621 (Table 1, Case 1) and PP457622 (Table 1, Case 2) (Additional File 1).

**Table 1:** Summary of case diagnostic assay results conducted at the ISU-VDL and PCV2/3 multiplex qPCR for PCV4 positive lymphoid tissue, lung, and spleen samples.

Case no	Site state	Age (wk)	Clinical signs	Histopathology	PCV4		Viral coinfections Ct					Bacterial coinfections	
					Tissue	Ct	PRRSV	IAV	PCV2	PCV3	Other (Ct)	Respiratory	Enteric
1	MO	16	Resp, systemic, diarrhea	IP, PE	LT	21.3	D	U	U	33.2	NP	SS*,* GP, BB	EC (U), LI
2	NE	F	Resp	No lesions	LT	25.6	NP	NP	18	U	NP	MHP (U)	NP
3	MN	3	Resp	IP	Lung	27	17.1	NP	28.7	U	NP	NP	NP
4	MN	16	Diarrhea	IP, PE	LT	30	NP	NP	U	U	NP	NP	LI
5	IN	NR	NR	IP	LT	31.8	17.3	U	34	36.9	NP	SE	NP
6	IA	5	Resp, death	Bronchiolitis, BP, SC	Spleen	32.2	U	27.9	U	U	NP	SS	EC*,* S. spp
7	IA	F	Resp	IP, bronchiolitis, polyserositis	Spleen	32.8	17.4	NP	17.7	30.8	NP	SS	NP
8	TX	3	Resp, death	IP, SC	Spleen	33	U	NP	U	U	NP	*SS*	EC
9	IA	6	Resp, CNS	No lesions	Spleen	33	NP	NP	U	U	NP	NP	EC, S. spp.
10	IN	G	Resp	BP, IN	LT	33	29.6	U	25.4	U	NP	MHP, PM	NP
11	IN	B	Resp	polyserositis	LT	33.6	U	U	U	33.1	NP	SS*,* GP	NP
12	IA	3	Resp, enteric	SC	Lung	34	U	U	33.2	U	PEDV (U), PDCoV (U), TGEV (U)	NP	NP
13	NC	3	Systemic, scours	Suppurative enteritis	Spleen	34	U	U	14.5	U	NP	NP	NP
14	IA	6	Resp, joint swelling	Bronchiolitis, BP, synovitis	Spleen	34	U	16.6	31	U	NP	SS	NP
15	IL	16	Resp, death	BP, polyserositis	LT	34	NP	32.8	29	22.3	NP	SS*,* GP, TP	NP
16	IN	23	Resp	IP, lymphoid depletion, myocarditis, IN	LT	34.1	U	U	22.2	U	NP	SE, PM	NP
17	IA	9	Resp, CNS	IP, BP, lymphocytic encephalitis	Spleen	34.2	15.2	NP	34.4	U	NP	PM	NP
18	IA	5	Diarrhea	Bronchiolitis, AE	Spleen	34.3	NP	27.2	U	U	Rotavirus A, B, C (D); sapovirus (D)	NP	EC
19	IN	16	Resp, death	IP, EM	Spleen	34.3	20.7	NP	U	U	NP	SS*,* SE	NP
20	IA	3	NR	NP	Lung	34.4	U	NP	36.2	U	NP	NP	NP
21	IA	16	Systemic, death	BP	Lung	34.4	23	NP	U	U	NP	AS	NP
22	MO	4	Resp, enteric	Bronchiolitis, BP, AE, SC	Lung	34.5	NP	21	U	29	NP	SS*,* SE	EC, S. spp
23	IN	8	NR	NP	LT	34.8	NP	NP	U	U	NP	NP	NP
24	IN	8	NR	NP	LT	34.9	NP	NP	U	U	NP	NP	NP
25	NC	17	Resp, death	Chronic epicarditis	Lung	35	18.9	U	D	U	NP	NP	NP
26	MO	12	Resp, systemic	IP, BP	LT	35.1	29.5	U	U	U	NP	SS	NP
27	NE	22	Death, purple discoloration of skin	IP, necrosuppurative BP	LT	35.2	NP	NP	U	U	NP	SE, AS	NP
28	IA	6	Enteric	Suppurative enterocolitis	Lung	35.4	NP	NP	34.3	U	NP	NP	EC
29	IA	12	Resp	IN, IP, BP, lymphoid depletion	Lung	35.4	positive	NP	9.4	U	NP	NP	NP
30	IA	4	Diarrhea	IP, BP, AE, myocarditis	Lung	36.2	positive	U	U	U	Rotavirus A (D)	SS	EC

Age abbreviations- B: Boars in breeding herd, F: Finishing aged pig, G: Gilts in breeding herd.

Site state abbreviations- MO: Missouri, NE: Nebraska, MN: Minnesota, IN: Indiana, IA: Iowa, TX: Texas, NC: North Carolina, IL: Illinois.

Clinical sign abbreviations- CNS: Central nervous system, Resp: Respiratory.

Histopathology abbreviations- IP: interstitial pneumonia, PE: proliferative enteritis, BP: bronchopneumonia, SC: suppurative colitis, IN: interstitial nephritis, AE: atrophic enteritis, EM: eosinophilic meningoencephalitis.

Molecular diagnostics abbreviations- D: Detected, NR: not reported, NP: not performed, U: undetected after cycle cut-off.

Viral abbreviations- PRRSV: porcine reproductive and respiratory syndrome virus, IAV: influenza A virus, PCV2: porcine circovirus 2, PCV3: porcine circovirus 3, PEDV: porcine epidemic diarrhea virus, PDCoV: porcine delta coronavirus, TGEV: transmissible gastroenteritis virus.

Bacterial abbreviations- SS: Streptococcus suis, BB: Bordetella bronchiseptica, GP: Glaesseralla parasuis, SE: Streptococcus equisimilis, MHP: Mycoplasma hyopneumoniae, PM: Pasteurella multocida type A, TP: Trueperella pyogenes, AS: Actinobacillus suis, EC: Escherichia coli; S. spp: Salmonella species, LI: Lawsonia intracellularis.

### PCV4 in situ hybridization

In addition to the tissues selected for PCV4 PCR evaluation (lung, spleen, lymphoid tissue, and fetal tissues), clinical submissions often include an array of tissues that are routinely evaluated for histopathology. Thus, in situ hybridization was performed on all formalin-fixed paraffin-embedded (FFPE) stored tissues in all PCV4 positive cases with Ct values of < 30. FFPE samples were pretreated according to the Advanced Cell Diagnostics (ACD) RNAscope 2.5 assay user manual (UM 322,452), with a target retrieval for 15 min at 98–102 °C and protease plus incubation for 30 min at 40 °C. PCV4 probes targeting the ORF2 gene of PP457621 (target region 2–677 base pairs, ACD 1,312,351-C1) were utilized to target viral mRNA. The specific sequence used to design each probe is intellectual property of the provider (ACD, Newark, CA, USA), and therefore, is not available for public access. Positive hybridization signals represent a metabolically active virus characterized by the PCV4 mRNA­encoding Cap protein. A PCV4 negative detection control was included by staining sections of lymph node negative for PCV4 by qPCR. The technique controls, to assess background signal, include a house keeping positive control probe of *Sus scrofa* peptidylprolyl isomerase B (PPIB) gene and a negative control probe of *Bacillus subtilis* dihydrodipicolinate reductase (dapB) gene. RNAscope 2.5 HD Reagent Kit (ACD, Newark, CA, USA) was used as per manufacturer recommendation.

### Immunohistochemistry characterization of inflammatory cells

Inflammatory cell characterization was performed on sections of the lymph node and small intestine (both tissue sections belong to Case 1, Table 1) by immunohistochemistry (IHC). T lymphocytes were stained with rabbit anti-human CD3 (Agilent, Santa Clara, California, USA) at 1:200 dilution, B lymphocytes were immunolabeled with rabbit anti-CD20 (Biocare Medical, Concord, California, USA) at 1:200 dilution, and macrophages/histiocytes were immunolabeled with rabbit anti-Iba-1 (Abcam, Cambridge, United Kingdom) at 1:500 dilution. All antibodies were incubated for 1 h at room temperature. After primary antibody incubation, tissues were incubated with anti-mouse (DISCOVERY, Anti-Mouse HQ, Roche, Basel Switzerland) or anti-rabbit secondary antibody (DISCOVERY Anti-Rabbit HQ, Roche, Basel, Switzerland) for 12 min at 37 °C and followed by chromogenic staining with Anti-HQ HRP (DISCOVERY Anti-HQ HRP, Roche, Basel, Switzerland) for 12 min at room temperature (DISCOVERY HQ HRP hapten-linked multimer detection System, Roche, Basel, Switzerland). Chromogenic staining was developed with DAB chromogen (DISCOVERY ChromoMap DAB RUO, Roche, Basel, Switzerland) and counterstained with hematoxylin for 8 min.

### Direct detection methods for PCV3, PCV2, PRRSV, and *Lawsonia intracellularis*

The detection of PCV3 by in situ hybridization was performed as previously described^27^. Briefly, specific probes targeting the specific reverse complementary nucleotide sequence of the PCV3 viral mRNA (2–1049 region of ORF1 gene, GenBank: HQ839721.1) (ACD, Newark, CA, USA), were used for viral confirmation. The RNAScope positive control probe Sc­PPIB (catalogue no. 428591), which targets the eukaryotic PPIB gene, and the RNAScope negative control probe DapB (Catalogue No. 310043) were designed and synthesized by ACD. FFPE tissue sections were deparaffinized and treated with hydrogen peroxide at room temperature for 10 min. The slides were hybridized using a hybridization buffer, and sequence amplifiers were added. The red colorimetric staining detects the PCV3 hybridization signal, and counterstaining utilized hematoxylin.

Direct detection of PCV2, porcine reproductive and respiratory syndrome virus (PRRSV) and *Lawsonia intracellularis* was performed by IHC. The PCV2 detection was performed under Leica Bond RX system (Lecia, Wetzlar, Germany). All FFPE tissues were deparaffinized in xylene, rehydrated in gradients of ethanol followed by peroxidase blocking step for 5 min (BOND Polymer Refine Detection System, Leica, Wetzlar, Germany). The primary antibody against PCV2 at 1:1000 dilution (Rabbit Polyclonal ORF-2, ISU, Ames, IA, USA) was incubated for 15 min at room temperature and followed by polymer incubation for 8 min, Diaminobenzidine (DAB) chromogen for 10 min and counterstained with hematoxylin (BOND Polymer Refine Detection System, Leica, Wetzlar, Germany). The detection of PRRSV and *Lawsonia intracellularis* was performed under Ventana DISCOVERY ULTRA platform (Roche, Basel, Switzerland). All FFPE were deparaffinized in xylene, rehydrated in gradients of ethanol followed by digestion with Protease I for *L. intracellularis* and Protease II for PRRSV (Dispase II, Roche, Basel, Switzerland) for 12 min at 37 °C and blocked with one drop of CM Inhibitor (Roche, Basel, Switzerland) for 8 min. A cocktail of equal concentration of SDOW17 and SR30 PRRSV monoclonal antibodies at 1:16,000 dilution (RTI, Brookings, SD, USA) was incubated for 27 min at 37 °C; and a monoclonal against *L. intracellularis* at 1:500 dilution (Bio-X Diagnostics, Rochefort, Belgium) were incubated for 28 min at 37 °C. After primary antibody incubation, tissues were incubated with anti-mouse secondary antibody (DISCOVERY Anti-Mouse HQ, Roche, Basel Switzerland) for 12 min at 37 °C and followed by chromogenic staining with Anti-HQ HRP (DISCOVERY Anti-HQ HRP, Roche, Basel Switzerland) for 12 min at room temperature (DISCOVERY HQ HRP hapten-linked multimer detection System, Roche, Basel Switzerland). Chromogenic staining was developed with DAB chromogen (DISCOVERY ChromoMap DAB RUO, Roche, Basel Switzerland) and counterstained with hematoxylin for 8 min. All samples were tested in duplicate and sections were controlled appropriately for PCV2, PRRSV and *L. intracellularis* using standard positive controls validated at the ISU-VDL.

### Statistical analysis

Differences in average PCV4 Ct values were analyzed by one-way ANOVA with Tukey’s correction for multiple comparisons with statistical significance set at an alpha value of 0.05 in GraphPad Prism (GraphPad Software, La Jolla, CA, USA) (https://www.graphpad.com). The Venn diagrams with proportional areas representing the differential proportion of PCV4/3/2 codetection in individual tissues were generated using the JMP® Pro 17.1.0 (SAS Institute, Cary, North Carolina, USA) (https://www.jmp.com/en_us/home.html).

## Results

### PCV4 was detected in different sample matrices

**Figure 1:**
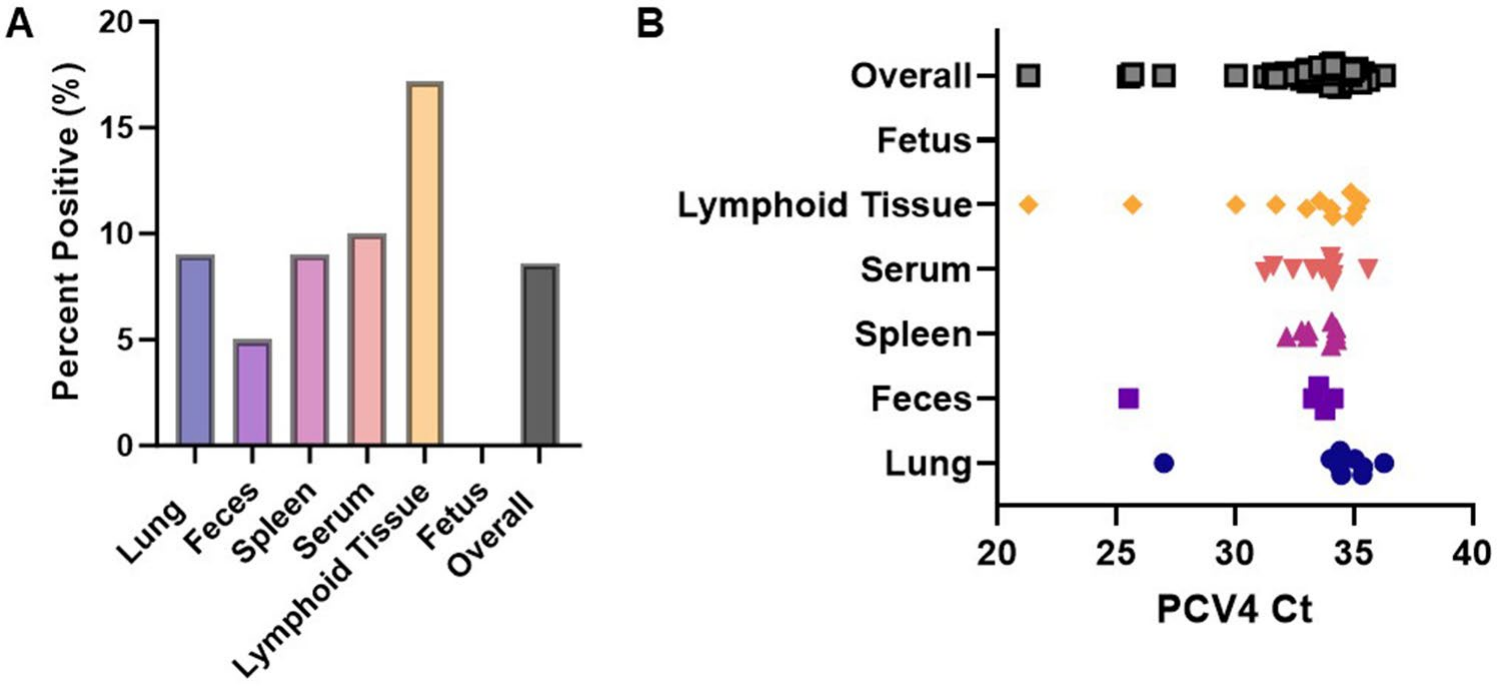
Detection of PCV4 from clinical cases submitted to the ISU-VDL from June–September 2023. Multiple sample types were evaluated for the presence of PCV4 by qPCR (**A**). Average distribution of Ct values amongst different sample types. No significant difference (*p* > 0.05) in the average Ct value was observed amongst different sample types (**B**). Significance was established at *p* < 0.05.

Overall, PCV4 was detected in 44 of the 512 samples (8.6%) [95% CI; 6.3–11.3] occurring in lung 9/100 [9%; 95% CI; 3.4–14.6], feces 5/100 (5%) [95% CI; 0.7–9.2%], spleen 9/100 (9%) [95% CI; 3.4–14.6%], serum 10/100 (10%) [95% CI; 4.9–17.6%], and lymphoid tissue 12/64 (18.7%) [95% CI; 9.1–23.8%] (Fig. 1A). Interesting, PCV4 was not detected in the fetus samples (0/48, 0%). The overall average PCV4 Ct value was 33, ranging from 21.3 to 36.2. Additionally, the average Ct value between different sample types was not significantly different (*p* > 0.05) (Fig. 1B).

### PCV4 open reading frame 2 sequencing and phylogenetic analysis

**Figure 2:**
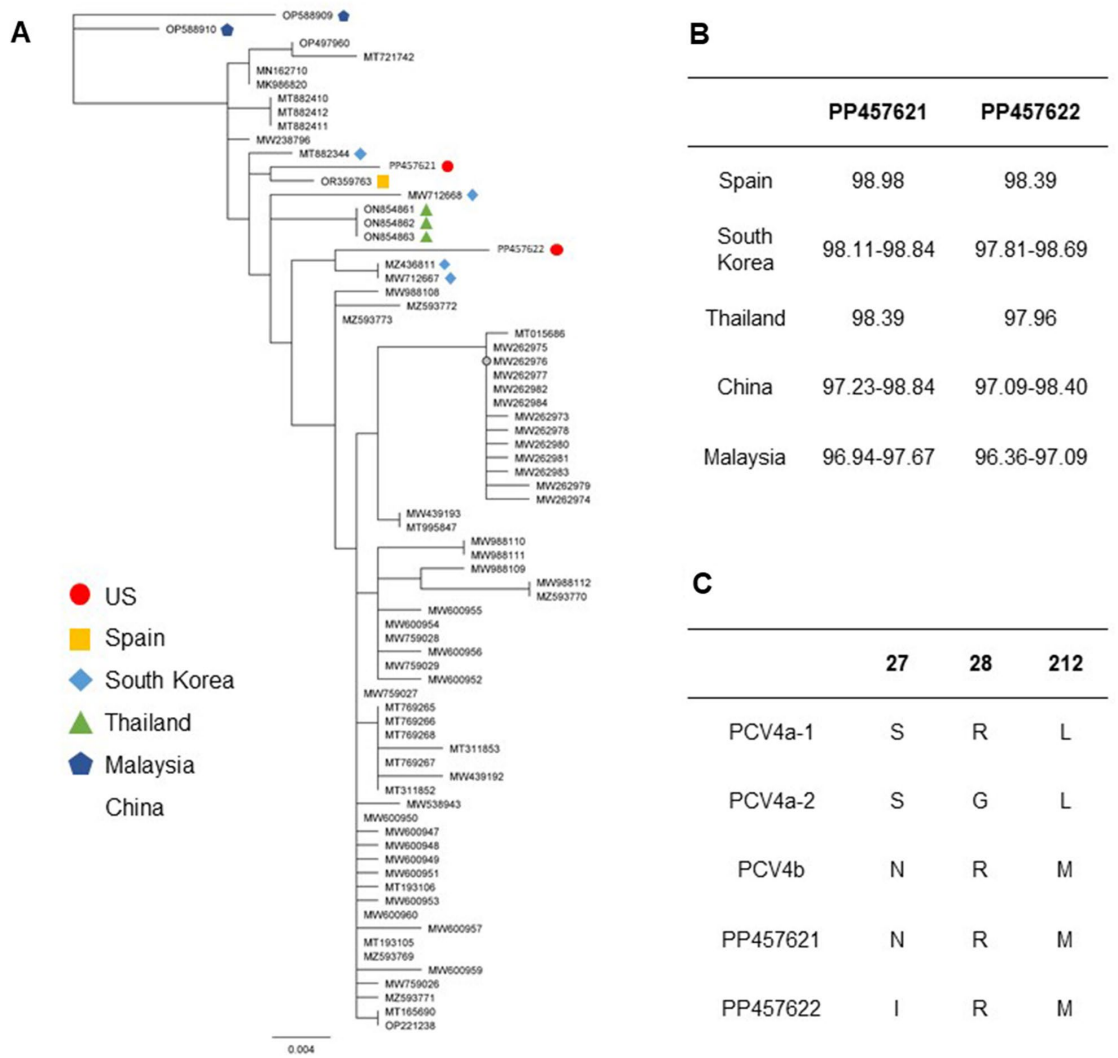
PCV4 ORF2 phylogenetic analysis and comparison of nucleotide percent identities. The phylogenetic tree of the two PCV4 ORF2 sequences obtained in the present study and 73 reference sequences was constructed using the maximum-likelihood method in Geneious Prime software with the bootstrap method of 1,000 replications (**A**). The range of nucleotide percent identities is summarized for the two US PCV4 ORF2 sequences in comparison to Spanish, South Korean, Thai, Chinese, and Malaysian sequences (**B**). ORF2 nucleotide sequences were translated using standard genetic code and were aligned using Clustal Omega in Geneious Prime. Comparison of PCV4 US ORF2 amino acids proposed for subtype classification (residues 27, 28, 212) to PCV4a-1, PCV4-a2, and PCV4b subtypes (**C**).

Two complete PCV4 ORF2 sequences were obtained from two positive lymphoid tissue samples (Table 1, cases 1 and 2) with a Ct value of 21.3 and 25.6, respectively. The two US sequences PP457621 and PP457622 had 98.25% nucleotide identity. Compared to the 73 reference PCV4 ORF2 sequences, overall nucleotide identity ranged from 96.36 to 98.98% for the two sequences obtained in the present study (Fig. 2B). Sequence PP457621 most closely clustered with the Spanish sequence OR359763 (98.98% identity) and sequence PP457622 most closely clustered with South Korean sequences MZ436811 and MW712667 (98.69% identity) (Fig. 2A,B). Nucleotide percent identities for the two sequences obtained in the present study had 98.39–98.98% identity with the Spanish sequence, 97.81–98.84% identity with the South Korean sequences, 97.96–98.39% identity with the Thai sequences, 97.09–98.84% identity with Chinese sequences, and 96.36–97.67% identity with Malaysian sequences (Fig. 2B).

Preliminary subtype classifications utilize primarily Cap protein residues 27, 28, 212 to characterize sequences into PCV4a-1, PCV4a-2, and PCV4b subtypes^18–20^. The amino acids at these specific positions for PP457621 are 27N, 28R, and 212 M and for PP457622 are 27I, 28R, and 212 M (Fig. 2C). Interestingly, the mutation S27I in PP457622 is a unique molecular feature which has not been previously identified in available reference sequences (Fig. 2C). Based on p-distance and Cap molecular features at residues 27, 28, and 212, both sequences in the present study most closely can be classified in the PCV4b subtype.

### PCV4 tissue distribution and immune cell characterization

**Figure 3:**
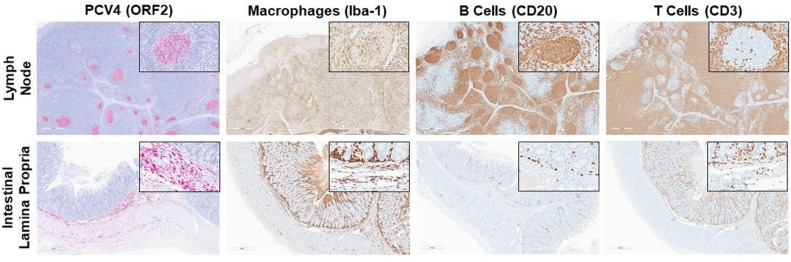
PCV4 in situ hybridization by RNAscope (red staining) targeting transcribed ORF2 mRNA revealed viral replication in the germinal centers of lymph node (top) and in inflammatory cells of the lamina propria of the small intestine (bottom). In the lymph node, CD20 B lymphocytes have a regular distribution in the germinal centers, and numerous macrophages (Iba-1) are present. Additionally, CD3 T lymphocytes are diffusely distributed in the paracortex and scattered detection is observed in the germinal centers. In the lamina propria, the predominant cells observed within this inflammatory response include macrophages (Iba-1) and CD3 T lymphocytes. A few resident CD20 B lymphocytes, located within the lamina propria, were also observed. Both sections of lymph node and intestinal lamina propria displayed in this figure were from Case 1 (Table 1).

Direct detection of PCV4 based on RNAScope (red signal) supports viral replication by detection of PCV4 mRNA in sections of lymph nodes and lamina propria of the small intestine (Fig. 3, Table 1 Case 1). Histologically, affected lymph nodes present diffuse paleness of the germinal centers with moderate histiocytic replacement (Supplementary Fig. 1). Subjective quantification of the immune cell populations by IHC revealed a more prominent histiocytic (Iba-1) infiltration of the germinal centers in addition to the resident B lymphocytes (CD20), which were located in areas associated with PCV4 infection (Fig. 3). However, the paracortex T cell population (CD3) did not overlap with the PCV4 staining (Fig. 3). Additionally, Case 2 (Table 1) also displayed direct detection of PCV4 in germinal centers of the lymph node (data not shown).

Direct detection of PCV4 based on RNAScope also demonstrated intense viral replication in the small intestine inflammatory cells of the lamina propria (Fig. 3, Table 1 Case 1). PCV4 replication was predominantly detected in areas with histiocytic (Iba-1) and T cell infiltration (CD3) of the lamina propria (Fig. 3). In contrast to the staining pattern observed in lymph nodes, PCV4 detection did not show significant overlap with areas characterized by resident B lymphocytes (CD20) (Fig. 3).

### Codetection of PCV4 with PCV2 and PCV3 by qPCR

**Figure 4:**
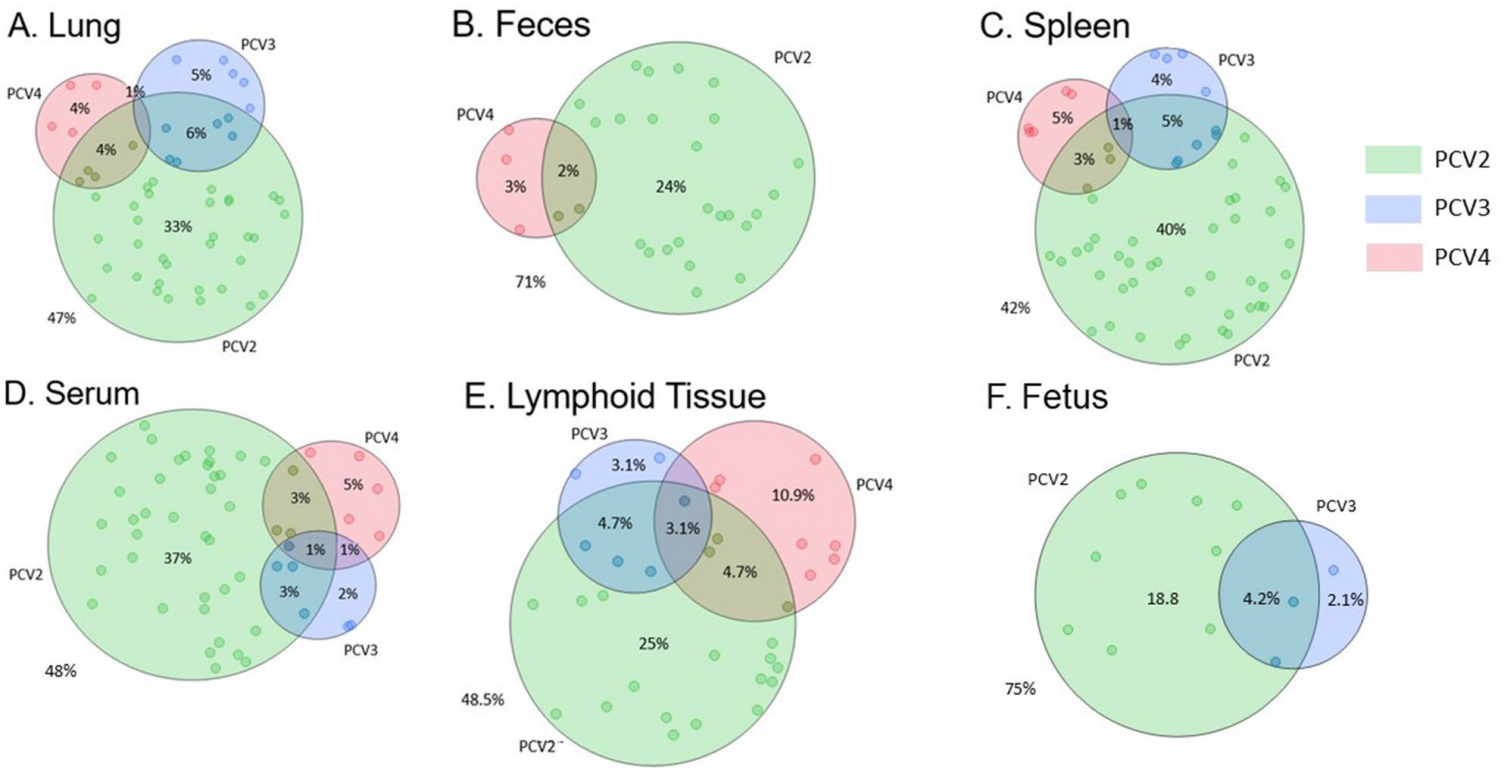
Codetection rate of PCV4 with PCV2 and PCV3 in lung (**A**), feces (**B**), spleen (**C**), serum (**D**), lymphoid tissue (**E**), and fetus (**F**). A total of 512 samples from clinical cases were tested by PCV4 singleplex qPCR and PCV2/3 multiplex qPCR. The Venn diagrams represent proportional areas with the differential proportion of PCV4/3/2 codetection (JMP® Pro 17.1.0; SAS Institute, Cary, NC).

**Figure 5:**
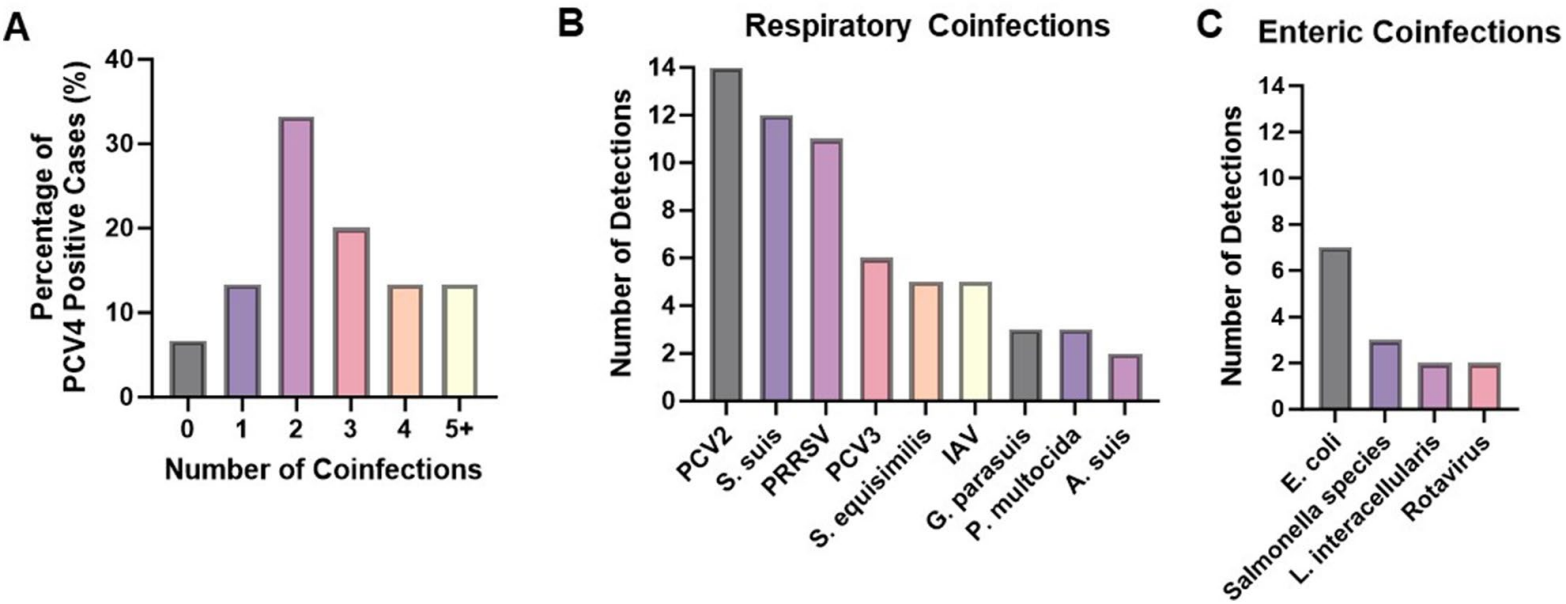
Summary of coinfections from ISU-VDL diagnostic case reports on cases with PCV4 detection in lymphoid tissue, lung, and spleen samples (*n* = 30). Percentage of PCV4 positive cases with 0–5 + coinfections identified in the diagnostic case report (**A**). Respiratory (**B**) or enteric (**C**) pathogens most commonly identified in PCV4 positive cases. Pathogens listed were identified in more than one PCV4 positive case.

The codetection of PCVs was assessed by PCV4 singleplex qPCR and PCV2/3 multiplex qPCR in all samples selected for study (Fig. 4A–F). Single detection of PCV4 and codetection of PCV4/2 were relatively similar for lung, feces, spleen, and serum sample types, ranging from 3 to 5% for PCV4 single detection and 2–4% for PCV4/2 codetection (Fig. 4A–D). However, lymphoid tissue displayed the highest detection rate for PCV4 single detection (10.9%), and PCV4/2 codetection (4.7%) (Fig. 4E). Codetection of PCV4/3 was relatively infrequent only occurring in 1% of lung and serum samples (Figs. 4A,D). Triple detection of PCV4/2/3 was also infrequent occurring in 1% of spleen and serum samples (Fig. 4C,D), but triple detection was more common in lymphoid tissue (3.1%) (Fig. 4E). PCV3 was not detected in fecal samples (Fig. 5B) and PCV4 was not detected in fetus samples (Fig. 4F). In samples with codetection, the average PCV2 and PCV3 Ct was 27.4 and 32, respectively. PCV2, PCV3, and PCV4 Ct values were not significantly different by sample type and codetection status (data not shown).

### Association of PCV4 with respiratory and enteric coinfections

**Figure 6:**
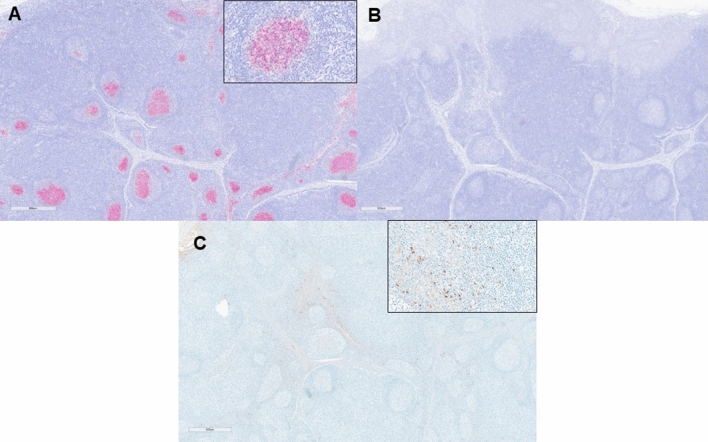
Direct detection of PCV4 in a PCV3/PRRSV coinfection case. PCV4 in situ by RNAscope (red staining) targeting transcribed ORF2 mRNA revealed viral replication in the germinal centers of lymph nodes (**A**). Meanwhile, the presence of PRRSV was confirmed by IHC, detecting viral antigen in the paracortical region of the lymph node (**C**). The presence of PCV3 was confirmed by qPCR; however, no PCV3 mRNA was detected in the lymph node by PCV3 RNAscope (**B**).

**Figure 7:**
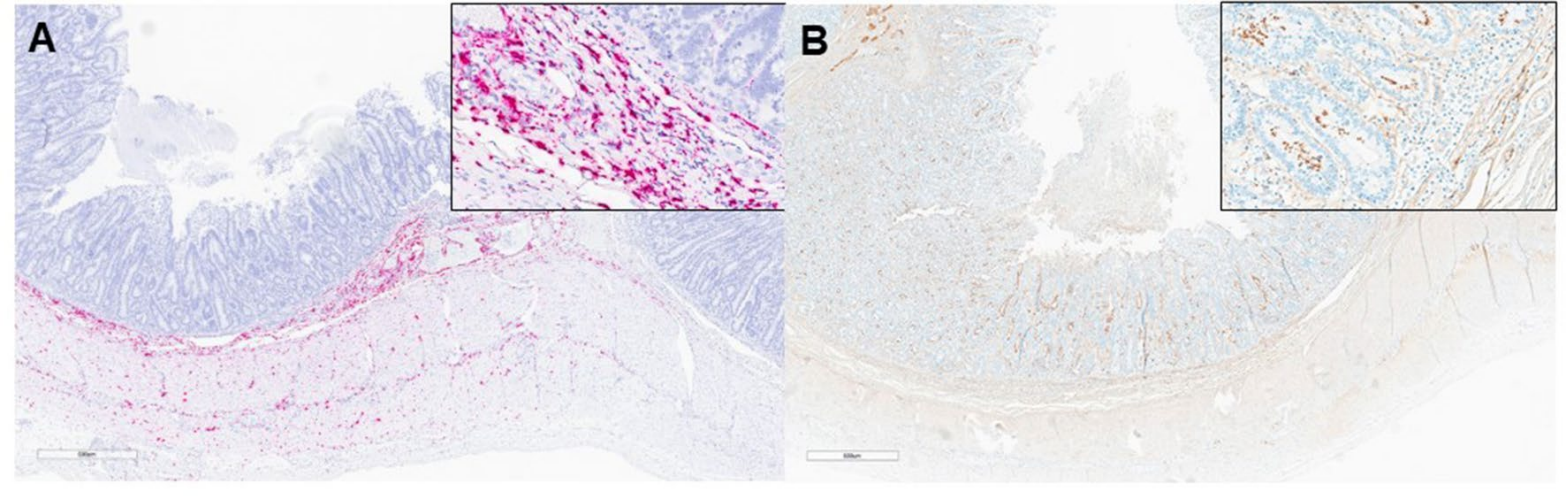
Direct detection of PCV4 in a case of *Lawsonia intracellularis*. PCV4 in situ by RNAscope (red staining) was confirmed within inflammatory cells of the lamia propria of the small intestine (**A**). Meanwhile, *L. intracellularis* was confirmed by IHC, displaying a characteristic staining pattern within enterocytes of the intestinal crypts (**B**).

The case results and information including age, location, clinical signs, histopathology, PCV4 qPCR, PCV2/3 multiplex qPCR, and diagnostic test results associated with the PCV4 positive lymphoid tissue, lung, and spleen samples (*n* = 30) are summarized in Table 1. Reported clinical signs in cases where PCV4 was detected most commonly included respiratory and enteric disease in 2–23-week-old pigs (Table 1). PCV4 was incidentally found in 28/30 (93.3%) cases (Fig. 5A). In respiratory cases where PCV4 was detected, nine additional respiratory pathogens were identified. The most common viral pathogens identified were PCV2, PRRVS, and PCV3 and *Streptococcus suis* was the most prevalent bacterial pathogen (Fig. 5B). In a case with confirmation of PCV4 (Table 1, case 1) by in situ hybridization (Fig. 6A), direct detection for PCV3 by in situ hybridization failed to confirm PCV3 mRNA in PCR-positive lymph nodes (Fig. 6B) while IHC demonstrated the presence of PRRSV antigen in paracortical macrophages and lymphocytes (Fig. 6C). In cases where enteric signs were the main clinical signs reported, the four enteric pathogens were identified. *Escherichia coli* and *Salmonella* species were the most commonly identified coinfections (Fig. 5C). In a case with enteric clinical signs and detection PCV4 in feces (Table 1, case 1), *Lawsonia intracellularis* direct detection by IHC revealed antigen signal in crypt enterocytes (Fig. 7B) while PCV4 detection by RNAScope was located in the inflammatory cells of the lamina propria (Fig. 7A).

## Discussion

PCV4 was initially identified in China in 2019^6^ and has subsequently been detected in Mongolia^7^, South Korea^8^, Thailand^9^, Malaysia^10^, and Spain^11^. Various detection methods have been utlized, including conventional or real-time quantitative PCR techniques, yielding variable detection rates ranging between 1.6% and 45.39%^7,12^. In the present study, PCV4 was detected for the first time in the US from clinical samples submitted to the ISU-VDL from June to September 2023. Samples evaluated in this study did not target a specific clinical syndrome and selection was based on random sampling of representative sample types used to evaluate different clinical syndromes. Thus, the overall positivity rate of this study (8.6%) falls within the range reported globally^18^. Interestingly, the detection rate varied by sample type for lymphoid tissue (17.2%), serum (10%), lung (9%), spleen (9%), and feces (5%), supporting the notion that the virus can be detected and may affect different organ systems. Relatively low amounts of PCV4 viral DNA were detected in most samples with an average Ct value of 33.0, which was not significantly different between sample types. Previous studies have reported the detection of PCV4 in a broad range of sample types including, lymph nodes, tonsils, serum, lungs, spleens, kidneys, hearts, livers, brains, intestines, and oral fluids^18^. In alignment with our results, lymph nodes have been identified with high detection rates of PCV4 ranging from 33.7 to 51.85% in different studies^11,12,19^. Several previous studies reported the detection of PCV4 viral DNA in aborted fetuses from sows with reproductive failure^8,12,28^, suggesting that PCV4 could be considered among the causes of reproductive failure in sows, abortion, mummies, and stillbirths^8,13^. However, our results, based on a relatively small sample size (*n* = 48), did not identify the presence of PCV4 in fetal tissue collected from a wide array of samples obtained from aborted fetuses, mummies, and stillbirths. Our results also align with similar results previously reported in an extensive study evaluating the presence of PCV4 in abortions and stillborn piglets in Colombia^14^. Reproductive failure in sows associated with PCV2 and PCV3 infection is characterized by abortion, mummies, and stillbirths. Furthermore, PCV2 and PCV3 can be detected by qPCR in addition to confirmation of viral replication by direct detection methods in the fetal tissues^28,29^. Thus, further studies are needed to characterize the presence and significance of PCV4 detection in tissue from aborted fetuses and stillbirths and confirm its potential role in reproductive failure. Based on our results, PCV4 tissue tropism may differ from PCV2 and PCV3, therefore potentially resulting in differences in clinical presentation and lesions associated with its infection.

Two full-length PCV4 ORF2 sequences were obtained from two PCV4 positive lymphoid tissues (Table 1, cases 1 and 2). Phylogenetic analysis was carried out with the two sequences from the present study and 73 full-length PCV4 ORF2 sequences obtained from GenBank, inclusive of sequences from Spain, South Korea, Thailand, Malaysia, and China (Additional File 1). The overall nucleotide identity of the two US sequences was relatively high with all reference sequences, ranging from 96.36 to 98.98%. US sequence PP457621 clustered with the Spanish sequence OR359763 (98.98% identity). Interestingly, OR359763 was obtained from a fecal sample from an Iberian pig located in mid-southwestern Spain collected in 2022^19^. US sequence PP457622 most closely clustered with South Korean sequences MZ436811 and MW712667 (98.69% identity). These South Korean sequences were obtained from oral fluid samples from commercial pigs in 2020^30^.

Proposed preliminary subtype classification is based on the genetic distance between subtypes as 0.05^20^ and amino acid patterns of the Cap protein at residues 27, 28, 212, yielding three subtypes: PCV4a-1 (27S, 28R, 212L), PCV4a-2 (27S, 28G, 212L), and PCV4b (27N, 28R, 212 M)^18–20^. Although the amino acids 28 and 212 for both sequences obtained in the present study were consistent (28R and 212 M), the sequences differed at residue 27. Sequence PP457621 contained the 27N mutation, aligning with previous classification systems for the PCV4b subtype. However, sequence PP457622 contained a unique S27I mutation, which has not been previously identified in PCV4 reference sequences. Based on the p-distance, sequence PP457622 can be classified as PCV4b. Given that evidence is lacking to support specific amino acid mutations result in biological differences, further research is needed to provide robust criteria for the PCV4 classification schemes as the number of PCV4 sequences increase. Several different PCV4 nomenclatures have been proposed which have not been standardized at the international level, potentially leading to confusing and misleading interpretations similar to what previously occurred with initial PCV2 and PCV3 classification systems^31–35^. Since, the PCV2 subtype definition is based on the following criteria: maximum within subtype p-distance of 13%, minimum cluster internal node bootstrap support of 70%, and at least 15 sequences identified^36^. PCV3 subtypes are defined based on bootstrap support (or posterior probability) > 0.9, maximum genetic distance of 3% and 6% at the complete genome and ORF2 levels, concordant results between ORF2 and the complete genome, and at least five sequences available^37^. Based on the limited number of sequences obtained in this study and the absence of correlation between the different subtypes reported and specific clinical presentations, this classification cannot be used to associate the role of different subtypes with various clinical manifestations. Additional studies are needed to evaluate whether amino acid mutations may lead to potential changes in immunodominant regions, affecting the host immune response and resulting in viral escape.

Multiple previous studies have described the codetection of PCV4 with PCV2 and PCV3 in several sample types^7,9,12,13,16,19,22–24^. In the present study, all samples were analyzed for the presence of PCV2, PCV3, and PCV4 viral DNA by qPCR. Single detection of PCV4 and codetection of PCV4/2 showed similar detection rates for lung, feces, spleen, and serum, which ranged between 3 and 5% for PCV4 single detection and 2 and 4% in samples with PCV4/2 codetection. Additionally, lymphoid tissue displayed the highest detection rate for PCV4 single detection (10.9%) and PCV4/2 codetection (4.7%). The PCV4/3 codetection was relatively infrequent and only confirmed in 1% of lung and serum samples. Thus, our results are consistent with previous studies indicating a higher prevalence of PCV4/2 codetection in comparison to the codetection of PCV4/3^12,13,22,23^. Previous reports have reported PCV2/3/4 triple detection with positive rates ranging from 0.36 to 14.47%^12,13,23,30^. In our current investigation, triple detection of PCV4/2/3 was infrequent, occurring in only 1% of spleen and serum samples. Although, triple detection was relatively more common in lymphoid tissue (3.1%). Most investigations assessing co-detection rates have been conducted with relatively modest sample sizes. Therefore, there is a pressing need for larger-scale studies encompassing a broader array of sample types to delineate the specific tissue types which are more frequently associated with the co-detection of multiple PCVs. Based on the present and previous results, coinfection with multiple PCVs is possible. Therefore, it is imperative to establish the pathogenic relationship between PCVs and determine if coinfection has a synergistic effect on disease expression.

In addition to reports documenting the coinfection of PCV4 with PCV2 and PCV3, PCV4 coinfection with pseudorabies, porcine epidemic diarrhea virus (PEDV), and PRRSV have been reported. These coinfections have been observed in pigs exhibiting lesions and clinical manifestations more closely associated with other PCVs, such as PDNS, PMWS, and reproductive failure. Furthermore, pigs with PCV4 coinfections have also been linked to a spectrum of nonspecific clinical signs, including respiratory, enteric, and neurologic signs^12,13,18,22–24^. In this study, samples were acquired from ISU-VDL cases which provides a comprehensive data set encompassing various diagnostic tests conducted and allows for determining the presence of coinfections. This approach facilitated a more thorough assessment of coinfection occurrences at the individual case level, thereby enhancing our understanding of coinfection dynamics. From the lung, spleen, and lymphoid tissue PCV4 positive samples (*n* = 30), the overwhelming majority of cases had at least one additional pathogen identified (93.3%) and cases most commonly had two pathogens identified (33.3%). Additionally, the most commonly reported clinical signs in PCV4 positive cases were respiratory and enteric disease in 2–23-week-old pigs. Accordingly, respiratory and enteric pathogens were the most common coinfections identified. Amongst respiratory cases with PCV4 detected, coinfections with PCV2, *Streptococcus suis,* and PRRSV were most frequently identified with 14, 12, and 11 detections, respectively. In an observed case of PRRSV and PCV4 codetection (Table 1, Case 1), direct detection of both pathogens was identified by direct detection in the lymph node, however, direct detection for both viruses was unsuccessful in the lung. Notably, PRRSV was localized in the paracortical region, while PCV4 was detected within germinal centers. Further investigation is needed to determine whether PCV4 predominantly replicates within resident macrophages or is disseminated to different anatomical locations by circulating histiocytes, thereby clarifying its direct involvement in potential lung lesions. Although data on the cellular and anatomical compartmentalization of PCV4 within the lung were unavailable, its detection in histiocytes within lymph nodes suggests the possibility of its presence in pulmonary macrophages, especially during an exacerbated histiocytic response characteristic of PRRSV infection. In this case, the absence of PCV4 direct detection in the lung may be attributed to a low viral load or the sensitivity limitations of the employed direct detection methods. Moreover, further investigation is warranted to evaluate whether this coinfection exacerbates pulmonary lesions associated with PRRSV or lymphoid lesions linked to PCV4 and to elucidate the intricate interplay between these pathogens in modulating the immune response. Additionally, the lack of direct PCV3 detection in the same case may be due to a low viral load, as indicated by Ct PCR values, and the sensitivity limitations of the employed detection methods.

*Escherichia coli* and *Salmonella* species were most commonly identified with 7 and 3 detections, respectively, amongst enteric cases. Anecdotally, one case demonstrated the presence of PCV4 and *L. intracellularis* coinfection within the small intestine confirmed by direct detection techniques. PCV4 was detected in the feces by qPCR and by RNAscope suggesting replication was occurring in areas overlapping with inflammatory cells in the lamina propria. Interestingly, *L. intracellularis* was also present in the small intestine as evidenced by antigen signal in crypt enterocytes by IHC. Coinfection with PCV2 and *L. intracellularis* has been documented in clinical cases^38^ and reproduced experimentally^39^. It has been speculated that *L. intracellularis* may boost the possibility of PCV2 replication, especially in acute cases. Therefore, this hypothesis should be considered and further evaluated in cases of enteric bacterial disease and PCV4. Taken together, these results confirm that PCV4 is often detected with other coinfections. Samples in this study were randomly selected, and no specific clinical syndrome or the presence of infection was a prerequisite for inclusion in this survey, which may partially explain the large number of cases with coinfections with relatively low amounts of PCV4 present. We emphasize that confirmed viral replication in tissue by RNAscope and detection by qPCR does not establish causation of clinical disease. Identifying PCV4 in tissues submitted for the identification of various clinical syndromes may suggest potential coinfection scenarios, and the role of PCV4 either as a primary or predisposing factor for clinical disease warrants further investigation.

Although the pathogenesis of PCV4 remains poorly understood and its role in clinical disease is still debated, the present study confirms viral replication through direct detection. Only one previous study has attempted to demonstrate viral replication by direct detection using in situ hybridization, which showed viral replication in respiratory epithelial cells and lymphocytes^9^. In this study, the use of RNAscope technology enabled the confirmation of viral replication as the probes target replicative ORF2 mRNA. Our study detected viral replication in the germinal centers of lymph nodes and inflammatory cells in the small intestine. The cellular characterization in the lymph node suggests that PCV4 may have a higher affinity for B lymphocytes (CD20) and macrophages (Iba-1). The presence of positive macrophages within germinal centers may indicate ongoing chronic inflammation or perhaps migration of antigen-presenting cells to the germinal centers. However, cellular characterization of the areas of inflammation in the small intestine revealed that PCV4 has a higher affinity for T lymphocytes (CD3) and macrophages (Iba-1). These two cell types are not resident populations of the intestinal lamina propria, suggesting that PCV4 may replicate within these inflammatory cells. Since there is an ongoing coinfection with *L. intracellularis,* we speculate that primary inflammatory processes induced by bacterial infection may exacerbate PCV4 local replication. The main limitation of this histological evaluation is the lack of co-localization of cellular markers and PCV4. While a co-localization could be more informative, based on staining of replicated sections targeting the same organ, it can be speculated that PCV4 consistently replicates in macrophages (Iba-1) and B lymphocytes (CD20) within lymph nodes and areas of inflammation. These results may also suggest a similar cellular tropism of PCV4 as previous studies have identified for PCV2, where replication efficiently occurs in the lymphoid tissues^40–43^. Macrophages rarely support PCV2 replication and act as a “Trojan horse” to carry virus throughout the body^44–47^. The lesions of PCV4 infection in the lymph node seem to more closely resemble PCV2 lymphoid depletion^3,9^ in contrast to multisystemic perivasculitis, which is more commonly associated with PCV3 infection^28^.

In conclusion, this study provides valuable insights into the frequency of detection, distribution, and genetic characteristics of PCV4 in the US. Since its initial identification in China in 2019, PCV4 has been detected in various countries in Asia and Europe and now the US. This study identified PCV4 in clinical samples submitted to the ISU-VDL, with an overall positivity rate similar to what has been reported in other countries. The detection rate varied across different sample types, emphasizing the importance of considering tissue tropism when evaluating PCV4 infection. Furthermore, phylogenetic analysis revealed relatively high nucleotide identity between US sequences and reference sequences from other countries, suggesting potential global dissemination of PCV4 strains. Current classification schemes based on genetic and amino acid variations require further standardization to enable accurate subtype classification. Coinfection with other pathogens, including PCV2 and PCV3, was also observed, highlighting the complex interplay between different PCVs and their potential roles in disease pathogenesis. Further research is warranted to elucidate the pathogenic mechanisms and clinical implications of PCV4 infection, and the interaction with PCV4 and other pathogens in pigs. This study underscores the importance of ongoing surveillance and research efforts to better understand and mitigate the impact of PCV4 infections on swine health and production.

### Supplementary Information

Supplementary Information.

## Data Availability

The datasets generated and analyzed during the current study are available in the NCBI genomes repository under the accession numbers PP457621 and PP457622.
