# Supplementary material for: First detection of PCV4 in swine in the United States: codetection with PCV2 and PCV3 and direct detection within tissues
Source: Sci Rep. 2024 Jul 5;14:15535. doi: 10.1038/s41598-024-66328-y (PMC11226432; doi:10.1038/s41598-024-66328-y)
Supplement: Supplementary file 1 — Supplementary Information. [file 41598_2024_66328_MOESM1_ESM.pdf]

Additional Data Table

PCV4 sequences used for phylogenetic analysis

GenBank / ID	Country	Collection Year
PP457621	USA	2023
PP457622	USA	2023
MW262975	China	2022
MW262973	China	2022
MW262976	China	2022
MW262977	China	2022
MW262978	China	2022
MW262982	China	2022
MW262984	China	2022
MW262980	China	2022
MW262974	China	2022
MW262979	China	2022
MW262981	China	2022
MW262983	China	2022
MT015686	China	2020
MW988111	China	2022
MW988110	China	2022
MW439193	China	2022
MT995847	China	2021
MW084633	China	2021
MZ593770	China	2022
MW988112	China	2022
MW988109	China	2022
MW600955	China	2021
MW600956	China	2021
MW600952	China	2021
MW759029	China	2022
MW759028	China	2022
MW600954	China	2021
MT193105	China	2021
OP221238	China	2022
MT165690	China	2020
MZ593771	China	2022
MW600950	China	2021
MW600959	China	2021
MW600957	China	2021
MW600960	China	2021
MW759027	China	2022

MZ593769	China	2022
MW759026	China	2022
MW600953	China	2021
MW600951	China	2021
MW538943	China	2021
MW600949	China	2021
MW600948	China	2021
MW600947	China	2021
MT193106	China	2021
MT311853	China	2020
MW439192	China	2022
MT769267	China	2020
MT769266	China	2020
MT769265	China	2020
MT769268	China	2020
MT311852	China	2020
MZ593773	China	2022
MZ593772	China	2022
MW988108	China	2022
MZ436811	South Korea	2021
MW712667	South Korea	2022
MW712668	South Korea	2022
ON854863	Thailand	2023
ON854862	Thailand	2023
ON854861	Thailand	2023
MT882344	South Korea	2021
MW238796	China	2022
MN162710	China	2019
MK986820	China	2019
OP497960	China	2022
MT721742	China	2021
MT882412	China	2021
MT882411	China	2021
MT882410	China	2021
OP588910	Malaysia	2023
OP588909	Malaysia	2023
OR359763	Spain	2023

Supplementary figure 1:

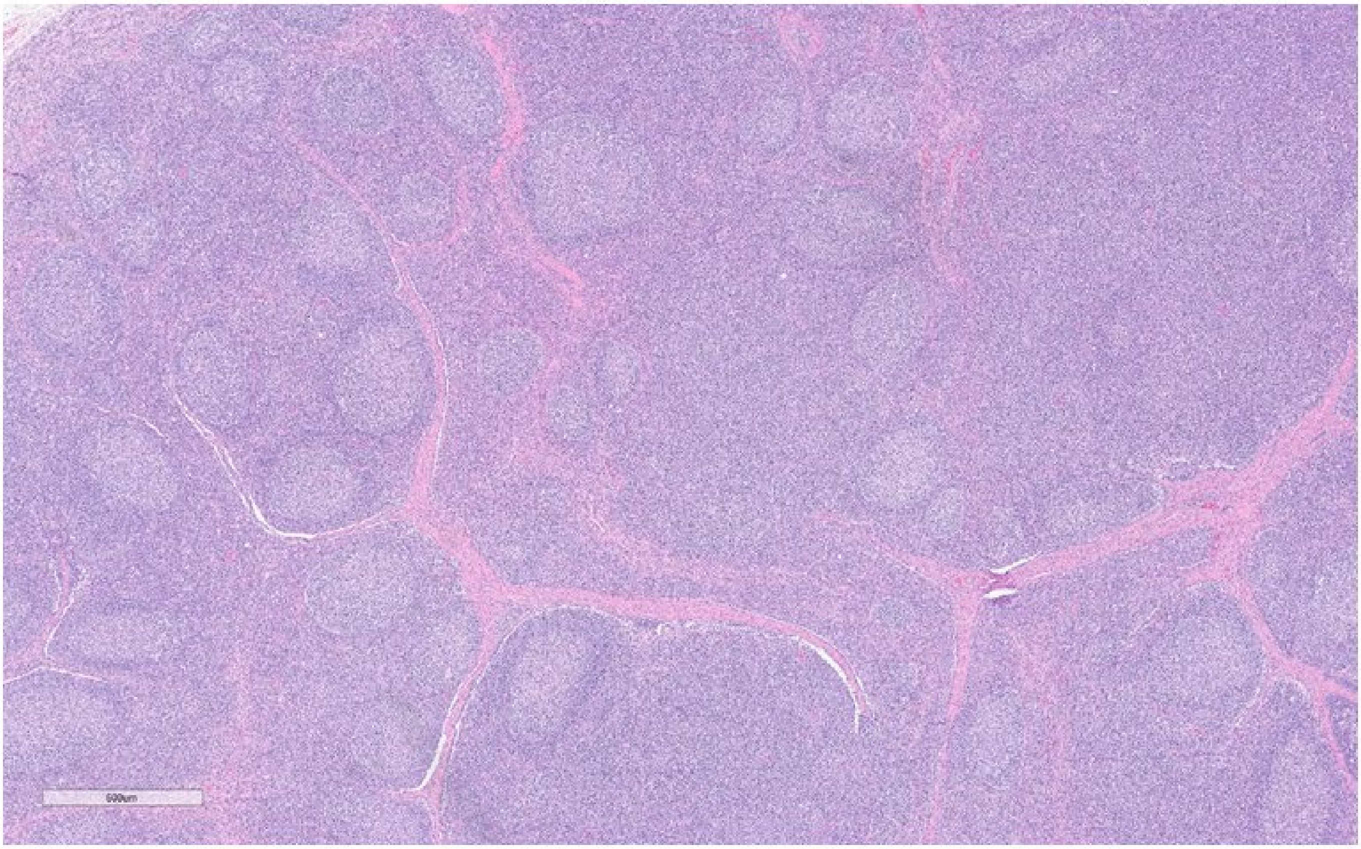

Supplementary figure1: Histological evaluation of affected lymph nodes presents diffuse paleness of the germinal centers with moderate histiocytic replacement.
